# GC/MS Fatty Acid Profile of Marine-Derived Actinomycetes from Extreme Environments: Chemotaxonomic Insights and Biotechnological Potential

**DOI:** 10.3390/md23010001

**Published:** 2024-12-24

**Authors:** Marlene B. Cunha, André F. Jorge, Maria João Nunes, Joana R. Sousa, Maria João Lança, Marco Gomes da Silva, Susana P. Gaudêncio

**Affiliations:** 1 Associate Laboratory i4HB, Institute for Health and Bioeconomy, NOVA School of Science and Technology, UNOVA University of Lisbon, 2829-516 Caparica, Portugal; mb_cunha@outlook.com (M.B.C.); jrl.sousa@campus.fct.unl.pt (J.R.S.); 2 UCIBIO—Applied Molecular Biosciences Unit, Department of Chemistry, NOVA School of Science and Technology, UNOVA University of Lisbon, 2829-516 Caparica, Portugal; 3 LAQV—Requimte and Department of Chemistry, NOVA School of Science and Technology, UNOVA University of Lisbon, 2829-516 Caparica, Portugal; af.jorge@campus.fct.unl.pt (A.F.J.); mjm.nunes@fct.unl.pt (M.J.N.); mdr@fct.unl.pt (M.G.d.S.); 4 MED—Mediterranean Institute for Agriculture, Environment and Development & CHANGE—Global Change and Sustainability Institute, Instituto de Investigação e Formação Avançada, Universidade de Évora, Pólo da Mitra, Ap. 94, 7006-554 Évora, Portugal; mjlanca@uevora.pt; 5 Departamento de Zootecnia, Escola de Ciências e Tecnologia, Universidade de Évora, 7006-554 Évora, Portugal

**Keywords:** marine actinobacteria, marine extreme environments, GC/MS fatty acids profiling, lipidomic FAMEs, oil extraction, microbial cell factories, blue biotechnology, circular bioeconomy

## Abstract

This study investigated the fatty acids (FA) profile of 54 actinomycete strains isolated from marine sediments collected off the Portugal continental coast, specifically from the Estremadura Spur pockmarks field, by GC/MS. Fatty acid methyl esters (FAMEs) were prepared from the ethyl acetate lipidic extracts of these strains and analyzed by gas chromatography–mass spectrometry (GC/MS), with FA identification performed using the NIST library. The identified FAs varied from C12:0 to C20:0, where 32 distinct FAs were identified, including 7 branched-chain fatty acids (BCFAs), 9 odd-chain fatty acids (OCFAs), 8 monounsaturated fatty acids (MUFAs), 6 saturated fatty acids (SFAs), 1 polyunsaturated fatty acid (PUFA), and 1 cyclic chain fatty acid (CCFA). The average expressed content was BCFA (47.54%), MUFA (28.49%), OCFA (26.93%), and SFA (22.16%), of which i-C16:0, C18:1ω9, and C16:0 were predominant, while PUFA (3.58%) and CCFA (0.41%) were identified as minor components. The identified BCFA were i-C16:0, a-C15:0, i-C15:0, i-C15:1ω6, a-C16:0, a-C14:0, and i-C17:0, which include combined branching and unsaturation and branching and odd. SFAs were present in all species, with C16:0 and C18:0 being the most representative. Rare OCFAs C19:1ω9, C17:1ω7, C15:0, and C17:0 were expressed. PUFA C18:1ω9 was detected; within this class, omega families ω9, ω7, ω6, and ω5 were identified, and no ω3 was detected. The only CCFA was benzene-butanoic acid (benzene-C4:0). These findings highlight the metabolic versatility of actinomycetes, providing valuable insights into microbial chemotaxonomy and offering promising biochemical leads for the development of biofuel, nutraceutical, and antifungal agents. Furthermore, these results underline the diversity and biotechnological potential of FAs in actinomycetes, uncovering their potential to be used as microbial cell factories, and paving the way for innovations in biofuels, pharmaceuticals, and eco-friendly industrial products.

## 1. Introduction

The adaptability of marine-derived actinomycetes (Phylum: Actinobacteria) endows them with unique metabolic and physiological capacities, including the production of novel bioactive metabolites [1,2,3,4]. These capabilities are closely associated with the presence of two key enzymes in their genomes, polyketide synthase (PKS) and non-ribosomal peptide synthase (NRPS), which are precursors for the majority of bioactive compounds [5]. These enzymes function through successive mechanisms of decarboxylative condensation of Acyl-CoA derivatives, which themselves serve as precursors for fatty acids (FAs) [6]. Actinomycetes are prolific producers of primary and secondary metabolites, making them highly valuable for diverse biotechnological applications with significant economic potential [7]. While research has traditionally focused on terrestrial actinomycetes, their marine counterparts, particularly those from extreme environments, remain comparatively underexplored [8,9].

Fatty acids are an important class of biomolecules. Most naturally occurring FAs have unbranched chains with an even number of carbon atoms, ranging from C4 to C28. Lipids containing esterified FAs, known as acyl lipids, are prevalent in marine environments and encompass both polar and non-polar types. The three major classes of naturally occurring FAs are triacylglycerols (TAGs), which consist of three FA molecules esterified to a glycerol backbone; wax esters (WEs), which consist of a FA esterified to a fatty alcohol; and phospholipids (PLs), which typically consist of two FA molecules esterified to a glycerol molecule that also contains a polar phosphatidic acid derivative.

FAs are vital dietary and energy sources for animals, as well as key structural components of cells. Vertebrates, including humans and fish, lack the Δ12 and ω3 (Δ15) desaturases required to convert C18:1ω9 (oleic acid) into essential polysaturated fatty acids (PUFAs) such as C18:2ω6 (linoleic acid, LA) and C18:3ω3 (α-linoleic acid, ALA). These PUFAs are precursors to highly unsaturated fatty acids (HUFAs) like C20:4ω6 (arachidonic acid, AA), C20:5ω3 (eicosapentaenoic acid, EPA), and C22:6ω3 (docosahexaenoic acid, DHA), which are essential for various physiological processes [10].

Microbial FA production offers a sustainable alternative to plant- and animal-derived FAs, with advantages such as high yield on diverse substrates, independence from seasonal constraints, and no requirement for large-scale cultivation areas [11]. Actinomycetes are particularly notable for their ability to produce FAs, which exhibit numerous bioactivities. These include antibacterial [5,12,13], antioxidant [5], antifungal [13,14], and cellulase activity [15]. This wide range of bioactivities highlights their potential for biotechnological applications, such as the development of pharmaceutical drugs [13,14] and their use in agriculture and aquaculture [15].

Previous studies by our research group using GC/MS revealed the presence of C16, C18, C20, and C22 FAs in two actinomycete strains isolated from sediments collected off the Madeira archipelago at depths ranging from 10 to 1310 m [9,16].

The actinomycetes used in this study were isolated from oceanic sediments collected from the Estremadura Spur pockmarks field off Portugal, located at depths of 220 to 350 m [17]. These strains were previously analyzed using LC-MS/MS Molecular Network annotation and Qemistree tools, aided by GNPS [18], to profile and dereplicate their secondary metabolites [19,20]. Surprisingly, this analysis revealed that strains of *Streptomyces* and *Micromonospora* produced significant amounts of FAs, indicating a specialized metabolism adapted for FA production [21]. Given the potential benefits and applications of FAs, the present study comprehensively examined the FA profile of actinomycetes from the Estremadura Spur using GC/MS.

## 2. Results and Discussion

### 2.1. Analysis of FAME Profiles of Actinomycetes Species Using GC/MS

**Figure 1:**
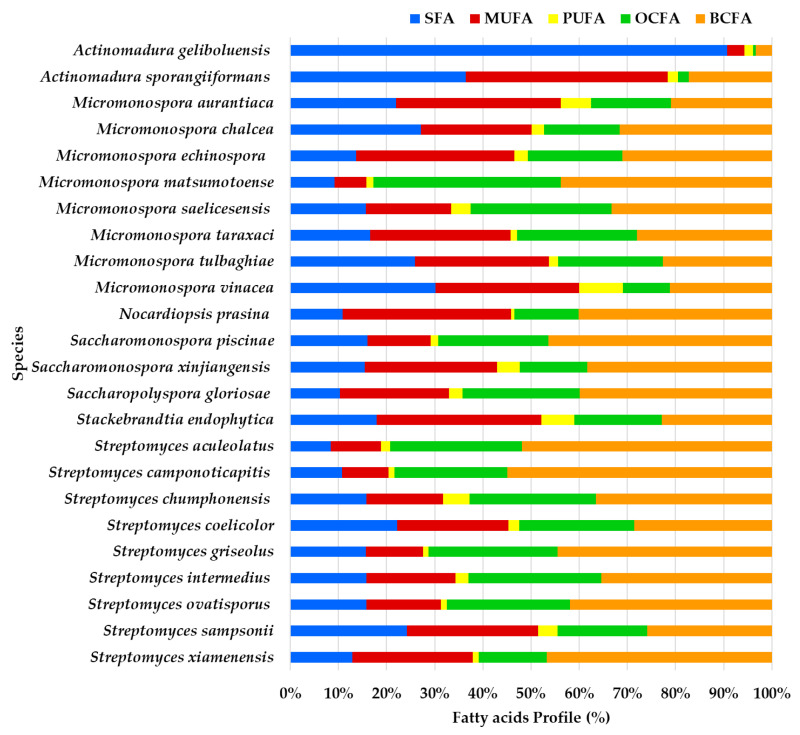
Profile of fatty acids’ main classes (expressed in %) from the ethyl acetate lipid extracts of the actinomycetes species isolated from marine sediments of the Estremadura Spur pockmarks.

The GC/MS data analysis provided insights into the FA profile of 54 supernatant ethyl acetate lipid extracts derived from actinomycetes species isolated from marine sediments collected in the Estremadura Spur pockmarks field (Portugal) (Figure 1, Table S1). Pockmarks are craters-like concavities on the ocean floor. When active, they exhibit hydrocarbons and gas seepage (e.g., CH_4_ and H_2_S), and are considered extreme marine environments known as cold seeps [4,17,22,23,24,25]. The taxonomic identification of these actinomycete strains was previously detailed in our study [21]. The 54 actinomycete strains used in this study represent 24 distinct species across 7 genera.

The analysis of FAME profiles from the crude extracts of the 54 actinomycete strains led to the identification of a total of 32 distinct FA. Among these, nine were odd-chain fatty acids (OCFAs), eight were monounsaturated fatty acids (MUFAs), seven were branched-chain fatty acids (BCFAs), six were saturated fatty acids (SFAs), one was a polyunsaturated fatty acid (PUFA), and one was a cyclopropane-containing fatty acid (CCFA). Hydroxylated FAMEs were not detected.

Peak identifications were based on previous work conducted by the team on different matrices, specifically the muscle FA profiles of sea lamprey [26] and largemouth bass [27], where a particular FAME, identified but not included in the standard mixtures, was only considered when the mass quality match exceeded 90%. In one of these earlier studies [26], a multidimensional GC/MS approach was employed, coupling polar and non-polar columns to minimize false positive identifications caused by the co-elution of isomers. By exploring polarity in the first dimension and volatility in the second, it was possible to deconvolute and tentatively identify isomers of specific carbon chain lengths. This method circumvented the need for target derivatization or additional sample preparation for identifying double bond positions and methyl group locations along the acyl chain. Building on these findings, in another study [27], a single polar column GC/MS system for peak identification was used. A 37-component FAME mix and a 26-component BAME standard mix (both from Supelco) were employed for reference. Data analysis was performed using Bruker MS Data Review 8.0 software, and the NIST library was utilized for FAME identification by comparing mass spectra and/or Linear Retention Indices (LRIs). Identification was assigned by matching calculated LRIs with standards and the available literature, as facilitated by the NIST Mass Spectral Search Program (2011). Despite using a single polar column, this approach allowed for accurate peak assignment while achieving shorter chromatogram run times compared to the multidimensional GC/MS method used in the sea lamprey study. It is well known that LRIs on polar phases are prone to variability [28]. When standards were unavailable, identifications were made based on probability regions, as described in the aforementioned studies [26,27]. The same methodology for peak assignment, using a single polar column, was applied in the current work to characterize the actinomycete extracts. LRIs for this study are provided in the Supplementary Materials, Table S2.

The distribution of FAMEs was observed within Rt ranging from 16 to 34 min, spanning from C12:0 to the longer chain FA C20:0. Figure S1 presents a representative GC/MS chromatogram of the FAME profile from the lipid extract of the actinomycete strain PTE-072.

To facilitate a better understanding of Figure 1, it is important to note that FAs are aliphatic, typically straight-chain monocarboxylic acids [10], which can be either linear or branched (with methyl or hydroxyl groups) [29], featuring a carboxyl group (COOH) at one end and a methyl group (CH_3_) at the other end [10,30]. The carboxyl group is reactive, often forming ester bonds with alcohol groups, such as glycerol, to create acylglycerols [30]. FAs are categorized based on their chemical structure. Saturated fatty acids (SFAs) contain no double bonds [10], monounsaturated FAs (MUFAs) have one double bond [10,30], and polyunsaturated FAs (PUFAs), possess two or more double bonds [30]. Among PUFAs, two key families, omega-3 (ω3) and omega-6 (ω6), are distinguished by the position of the first double bond relative to the terminal methyl group [10,31,32]. Additionally, FAs can be classified as odd-chain fatty acids (OCFAs) if their carbon chains have an odd number of carbon atoms, or as branched-chain FAs (BCFAs), which are primarily SFAs with one or more methyl branches on the carbon chain.

BCFA constitutes the largest proportion of the FA profile in most species, particularly in *Streptomyces camponoticapitis*, *S. aculeolatus*, *S. xiamenesis*, and *Saccharopolyspora piscinae*. This suggests these species heavily utilize branched-chain fatty acids. SFAs also play a significant role in some species. For instance, *Actinomadura sporangiformans*, *A. geliboluensis*, and *Micromonospora chalcea* show a noticeable proportion of SFAs. MUFAs are significant in a few species, such as *Nocardiopsis prasina* and *Actinomadura sporangiiformans*. PUFAs are less prominent but are evident in some species like *Micromonospora vinacea* and *Stackebrandtia endophytica*. Odd-chain fatty acids (OCFAs) are significant in species such as *Micromonospora matsumotoense* and *Streptomyces intermedius*.

A greater similarity in the FAME profile was observed between *Streptomyces intermedius* and *Micromonospora saelicesensis*, as well as between *Stackebrandtia endophytica* and *Micromonospora aurantiaca*. In contrast, the greatest variation was noted between the FAME profiles of *Actinomadura geliboluensis* and *Micromonospora matsumotoense*. Less variation was observed among OCFAs and BCFAs compared to other FA classes, which is expected since only one of each of these FAs was identified. The greatest discrepancy in FA content was found in *Actinomadura geliboluensis*, which exhibited low content of BCFAs, OCFAs, PUFAs, and MUFAs, with a predominance of SFAs. Similarly, *Actinomadura sporangiiformans* revealed low OCFA and BCFA content.

FAs are essential for human health, playing a critical role in regulating biological functions and controlling various diseases [10,32]. Due to their beneficial properties, FAs are widely used in several industrial sectors, including food, chemicals, pharmaceuticals, cosmetics, nutraceuticals, biodiesel, and aquaculture [11,33]. The general biotechnological applications of FAs are summarized in Table 1.

A detailed discussion of the FAME types by actinomycete species will be provided in Section 2.1.1, Section 2.1.2, Section 2.1.3, Section 2.1.4, Section 2.1.5, Section 2.1.6 and Section 2.1.7, while a general overview of the FA classes by actinomycete genera will be presented in Section 2.2.

#### 2.1.1. Saturated Fatty Acids (SFAs)

**Figure 2:**
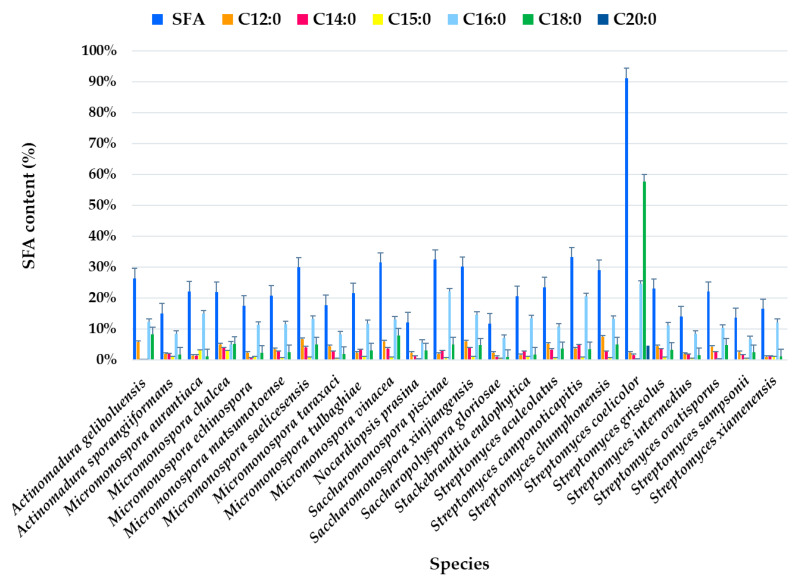
Individual SFA profile of the total SFAs (%) identified in ethyl acetate lipid extracts by actinomycetes species isolated from marine sediments of the Estremadura Spur pockmarks.

Analysis of the FAME profile revealed that the highest SFA content (>25% of the total FAMEs) was observed in *Actinomadura geliboluensis* PTE-033 (91.19%), followed by two *Micromonospora chalcea* strains PTE-068 (45.76%) and PTE-037 (40.56%), *Actinomadura sporangiiformans* (37.37%), *Micromonospora chalcea* (31.86%), *Micromonospora vinacea* (31.55%), *Streptomyces coelicolor* (30.14%), *Micromonospora tulbaghiae* (29.98%), *Streptomyces sampsonii* (29.81%), and *Micromonospora aurantiaca* (26.37%) (Figure 2, Table S1). However, a significant SFA content was observed in *Micromonospora chalcea* strain PTE-076 (9.26%), which is a notable outlier within this species. Similarly, variations in SFA content were found in other species, such as *Micromonospora saelicesensis* strains PTE-038 (34.83%) and PTE-083 (8.81%), and *Saccharopolyspora gloriosae* strains PTE-075 (5.69%) and PTE-077 (18.68%) (Figure 2). Although the literature suggests that each bacterial species has a unique FA profile, acting as a “fingerprint,” standardized growth conditions, such as culture medium, incubation temperature, and physiological age, can yield reproducible cellular FA profiles and enhance the accuracy of species identification [44]. Our study indicates that variations in FA production may still occur within the same species, even under identical experimental conditions. In 2019, our group also reported variations in the hybrid isoprenoid production among six *Streptomyces aculeolatus* strains from the Madeira Archipelago, despite using the same experimental conditions, highlighting intra-species metabolic variations [45,46,47].

SFAs were present in all species at levels >10% of the total identified FAs, except for *Saccharopolyspora gloriosae* PTE-075 (5.69%) and *Micromonospora chalcea* PTE-076 (9.26%) (Figure 2).

The most abundant FA was C18:0, which accounted for 57.65% of the total FAMEs in *Actinomadura geliboluensis*, followed by C16:0 (24.64%) (Figure 2). Together, these two FAs represented 63.22% and 27.02% of the total SFAs, respectively. According to our results, C16:0 was the predominant SFA in all species, except in *Actinomadura geliboluensis*, where C18:0 predominated. This result aligns with the literature, as C16:0 is characteristic of actinomycetes [48]. To the best of our knowledge, the predominance of C18:0 in *Actinomadura geliboluensis* has not been previously reported. In contrast, C18:0 was only identified as a minor component of total FAs in earlier studies of this species [49]. These discrepancies may be due to factors such as experimental conditions or the source of isolation [50]. However, *Actinomadura geliboluensis* is still understudied in terms of metabolite and FA production. A reported study of this species, isolated from soil in Çanakkale, Turkey, identified the major FAs (>10%) as i-C16:0 (19.93%), C17:1ω9 (16.53%), C16:0 (13.28%), C15:0 (11.60%), and 10-methyl C17:0 (11.59%), showing a lower proportion of C16:0 compared to our findings [49]. The predominance of C16:0 has been reported in other Actinomadura species, including *Actinomadura geliboluensis* (13.28%) [49], *Actinomadura jiaoheensis* (31.0%), *Actinomadura sporangiiformans* (21.2%) [51], *Actinomadura syzygii* (21.70%), *Actinomadura decatromicini* (23.10%), and *Actinomadura chibensis* (27.2%) [52], suggesting that C16:0 is characteristic of the *Actinomadura* genus [52].

Regarding longer-chain SFAs, C20:0 was only detected in *Actinomadura geliboluensis* (4.71% of total FAMEs, corresponding to 5.17% of total SFAs). Additionally, C12:0 (2.52%) and C14:0 (1.79%) were the less abundant SFAs. However, C14:0 was present in all species except *Micromonospora aurantiaca* (Figure 2).

The primary functions of SFAs include serving as an energy source and contributing to the structural integrity of cell membranes. A literature search on SFAs from marine-derived actinomycetes retrieved studies that describe their antifungal activity. The biological activity of extracts produced by marine-derived actinomycetes has shown that SFAs, particularly C14:0 and C16:0, exhibit antifungal properties, suggesting potential biotechnological applications, particularly in the food industry, where SFAs are used as food additives, emulsifiers, and lubricating and anti-foaming agents [14]. The antifungal activity of SFAs may also be useful for food preservation [34]. The mechanism of action of these FAs was reported, in which SFAs with antifungal activities penetrate the fungus cells’ membrane, leading to increased cell permeability, which results in the release of intracellular components, destroying their functions and, consequently, resulting in the cells’ death. Specifically, C14:0 has affinity for the enzyme N-myristoyltransferase 1 (NMT 1: EC 2.3.1.97), a key enzyme involved in lipid modifications that facilitates the attachment of myristate to the N-terminal glycine of various proteins. This enzyme is crucial for the growth and development of many eukaryotic organisms, fungi, and certain rotaviruses [53,54,55,56,57]. C14:0 binds to NMT1 through a process known as myristoylation (binding of myristic acid to the enzyme’s N-terminus), which alters the enzyme’s structure and function [14,58]. This inhibition can help prevent diseases caused by bacteria and viruses that rely on NMT1 for growth [58]. SFAs are known for their good oxidative stability [35]. Due to this property, along with other advantages such as a high cetane number and lower emissions of toxic gases, SFAs are also utilized in biofuel production [59].

The SFAs found in the actinomycete strains, including palmitic acid (C16:0) and stearic acid (C18:0), have significant industrial applications due to their oxidative stability and functional properties. These SFAs are valuable in biofuel production, food preservation, and antifungal formulations. Myristic acid (C14:0), in particular, has potential in combating pathogenic fungi and viruses by inhibiting the enzyme NMT1. High-SFA-producing strains like *Actinomadura geliboluensis* show promise for tailored applications in bioenergy, pharmaceuticals, and industrial additives. Further optimization of production conditions could enhance their commercial potential.

#### 2.1.2. Monounsaturated Fatty Acids (MUFAs)

MUFA content varies significantly among the actinomycete species, with some species having very high MUFA levels (around 30–50%). These were identified in all samples at a relatively high percentage (>10%), except for samples PTE-033 (3.69%) and PTE-083 (5.86%). The highest MUFA content was observed for *Streptomyces sampsonii* PTE-051 (49.24%). The biological activity of this strain demonstrated moderate inhibitory activity against *Staphylococcus aureus*, with a minimum inhibitory concentration (MIC) of 125 µg/mL [21].

**Figure 3:**
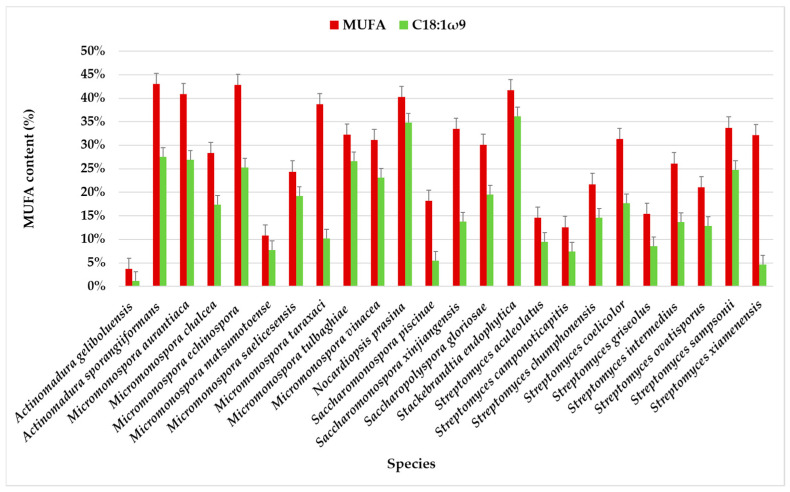
MUFA content in the total profile of FAMEs in actinomycete species isolated from marine sediments of the Estremadura Spur, with emphasis on C18:1ω9. Values expressed in % in relation to the total identified FA.

In terms of FA profiles, MUFAs accounted for the second-largest proportion, averaging 28.49% of the total FAMEs. As observed with saturated fatty acids (SFAs), considerable variations were detected within the same species. For example, in *Micromonospora saelicesensis* strains, the MUFA content was 42.95% in PTE-038 and 5.86% in PTE-083. Similar variations were observed among other species, with the lowest MUFA content (<10%) in *Actinomadura geliboluensis* (3.69%), and the highest content (>40%) in *Actinomadura sporangiiformans* (43.07%), followed by *Micromonospora echinospora* (42.81%), *Stackebrandtia endophytica* (41.70%), and *Micromonospora aurantiaca* (40.92%) (Figure 3, Table S1).

There is a significant variation in MUFA content among species of the genus *Actinomadura*. As discussed in the previous section, each bacterial strain has a unique fatty acid profile, which can serve as a chemotaxonomic marker when grown under controlled conditions (e.g., culture medium, temperature). However, changes in growth conditions, such as pH, salt concentration, time, and cell age, can lead to shifts in fatty acid composition, allowing the bacteria to adjust the fluidity of their membranes [44]. This suggests that the observed discrepancies in fatty acid profiles may stem from intra-species metabolic variations, as seen in our previous work with six *Streptomyces aculeolatus* strains, where different hybrid isoprenoid production was observed despite identical growth and extraction conditions [45,46,47].

The chromatographic analysis identified eight MUFAs, with C18:1ω9 being the most abundant, followed by C16:1ω7. C18:1ω9 accounted for more than half of the identified MUFAs in the species, except for *Streptomyces xiamenensis*, *Micromonospora taraxaci*, *Saccharomonospora xinjiangensis*, *Saccharomonospora piscinae*, and *Actinomadura geliboluensis*. The lowest content of C18:1ω9 (1.20%) was found in *Actinomadura geliboluensis* (Figure 3), consistent with a previous study where actinomycetes were isolated from soil [49]. The predominance of C18:1ω9 in *Stackebrandtia endophytica* (36.15%) and *Nocardiopsis prasina* (34.83%) contrasts with the previous study, where the content of C18:1ω9 in these species accounted for 2.40% [60] and 4.40% [61] of the total FAMEs, respectively. However, the high content of C18:1ω9 in *Nocardiopsis prasina* is consistent with the literature, as this genus is known to produce high levels of C18:1ω9 [62]. Conversely, *Stackebrandtia endophytica* is recognized for its high BCFA content [63]. Notably, a high content of C18:1ω9 (>25%) was also observed in *Actinomadura sporangiiformans* (27.53%), *Micromonospora aurantiaca* (26.97%), *Micromonospora tulbaghiae* (26.63%), and *Micromonospora echinospora* (25.32%), which has not been previously reported.

C16:1ω7 was present in all species, albeit in lower amounts in *Streptomyces ovatisporus* (0.59%). This species has been reported to produce high levels of BCFA [64]. The highest C16:1ω7 content was observed in *Micromonospora taraxaci* (25.18% of the total FAMEs), representing 64.98% of the total MUFAs, which contrasts with another study [65].

The least abundant MUFAs were C14:1ω5, C19:1ω9, C17:1ω7, and C16:1ω9. C14:1ω5 was identified in only three species: *Streptomyces xiamenensis* (0.37%), *Streptomyces intermedius* (0.54%), and *Actinomadura sporangiiformans* (2.21%) of the total MUFAs. Although C14:1ω5 is not common in actinomycetes, it is frequently found in fish oil and symbiotic bacteria [38,66]. This FA has also been identified in *Calidifontibacter* strains isolated from soil, although in low concentrations [67].

Interestingly, C19:1ω9 was only detected in the PTE-037 strain of *Micromonospora chalcea* (6.44%). Although its percentage was not high, it was the fifth most abundant fatty acid in this sample. C19:1ω9 is rarely found in actinomycetes, but it has been identified as the predominant component in the *Microcella* genus, which was recently isolated from marine sediments [68].

The highest content of C16:1ω9 was found in *Actinomadura sporangiiformanse* (10.13%). In contrast, it was relatively low in *Streptomyces aculeolatus* (0.18%) and absent in *Streptomyces camponoticapitis*, *Micromonospora chalcea*, *Nocardiopsis prasina*, *Micromonospora echinospora*, *Micromonospora tulbaghiae*, *Stackebrandtia endophytica*, *Saccharomonospora Piscinae*, and *Micromonospora aurantiaca*. This result aligns with expectations, as C16:1ω9 is a marker for Gram-negative bacteria [69].

The only identified complex FAME with both branching and unsaturation was i-C15:1ω6. This FA was absent in most species, and when detected, it was present in low amounts (<10%). The highest content was observed in *Streptomyces xiamenensis*, where it represented 10.23% of the total FAMEs. i-C15:1ω6 has only been identified in the genera *Streptomyces*, *Saccharomonospora*, and *Micromonospora*. Complex fatty acids are characteristic of the genus *Micromonospora* [70], although the present study found the lowest i-C15:1ω6 content in this genus.

The MUFA i-C15:1 has been identified in *Georgenia* and *Streptomyces*, although the exact position of the double bond remains unknown [71,72].

Among MUFAs, special emphasis is given to oleic acid (C18:1ω9), due to its demonstrated beneficial effects on human health, particularly in cardiovascular disease and atherosclerosis [73]. This FA has been widely used in biomedical applications, such as the synthesis of nanoparticles, owing to its ability to form a dense and protective monolayer [37]. Additionally, C18:1ω9 has application in the pharmaceutical, food, and cosmetic industries [74]. Palmitoleic acid (C16:1ω7) also has health and well-being benefits [73], such as promoting skin repair and regeneration, improving oxygenation, and facilitating toxin removal [75]. Furthermore, cis-9-heptadecenoic is an MUFA used in cosmetics and pharmaceuticals for the treatment of psoriasis, allergies, and autoimmune diseases [36].

The high MUFA content in the actinomycete strains, particularly oleic acid (C18:1ω9) and palmitoleic acid (C16:1ω7), highlights their industrial potential. Oleic acid’s cardiovascular benefits and use in pharmaceuticals, cosmetics, and food make it a prime target for commercialization. Palmitoleic acid’s skin repair properties further expand its application in cosmetics and wellness. Rare MUFAs like cis-9-heptadecenoic acid and i-C15:1ω6 offer niche uses in treating autoimmune diseases and psoriasis. The actinomycete strains identified in this study represent a sustainable and versatile resource for industrial production of MUFAs with wide-ranging applications in health, food, cosmetics, and advanced material synthesis.

#### 2.1.3. Polyunsaturated Fatty Acid (PUFAs)

Regarding PUFAs, the only one identified was linoleic acid (LA), C18:2ω6. The highest PUFA content was observed in the samples PTE-058 (19.33%) and PTE-078 (13.79%), corresponding to the species *Streptomyces chumphonensis* and *Saccharomonospora xinjiangensis*, respectively. On average, C18:2ω6 was relatively high in *Micromonospora vinacea* (9.79%), followed by *Stackebrandtia endophytica* (8.38%), and lower in *Nocardiopsis prasina* (0.82%). Interestingly, while C18:2ω6 was present in all samples, it was absent in two *Nocardiopsis prasina* strains (out of three), PTE-047 and PTE-048. According to the literature, this type of FA is uncommon in bacteria [76], particularly in actinomycetes. Nonetheless, it has been identified in *Mycobacterium hubeiense* [77], *Arcanobacterium pinnipediorum* [78], *Arcanobacterium wilhelmae* [79], and *Catellatospora sichuanensis* [80].

PUFAs are associated with the prevention and treatment of various diseases [81], including coronary heart disease [82,83], diabetes, multiple sclerosis, depression, and inflammatory conditions [84], such as asthma [83], rheumatoid arthritis [85], and psoriasis [86]. They play an important role in preventing and treating skin disorders like inflammation and aging [38]. In cosmetics, PUFAs are used for their moisturizing, photoprotective, and therapeutic properties [38]. Moreover, they are linked to the prevention of other conditions, such as cancer [87], Alzheimer’s disease [86], obesity, and kidney diseases [85]. PUFAs are particularly crucial during developmental phases in children, supporting eye and central nervous system development [86]. Additionally, PUFAs exhibit antibacterial, antifungal, and antioxidant properties, making them valuable in pharmaceutical, nutraceutical [33], cosmetic [38,74], and aquaculture applications [40,88].

The LA C18:2ω6 is recognized for its anti-inflammatory, immunomodulatory, anti-obesity, and anti-carcinogenic properties, as well as its ability to enhance cardiovascular health [89,90]. Additionally, marine fish typically exhibit low levels of LA and alpha-linolenic acid (ALA) but are rich in highly unsaturated fatty acids (HUFAs), such as eicosapentaenoic acid (EPA) and docosahexaenoic acid (DHA) [91,92,93]. These marine fish obtain PUFAs directly through their diet, as their ability to elongate and desaturate PUFAs has diminished due to the reduced activity of the enzymes involved in these processes [94,95]. Consequently, most marine species have low or insufficient capacities to convert ALA and LA into longer-chain fatty acids like EPA, DHA, and arachidonic acid (AA) [94,96]. In contrast, freshwater fish have higher levels of PUFAs with 18-carbon chains (e.g., LA and ALA) and appreciable amounts of AA, EPA, and DHA [91]. This difference is linked to their greater ability to elongate and desaturate fatty acids present in their diet, as they retain enzymatic mechanisms (e.g., Δ5 and Δ6 desaturases) for HUFA synthesis, compensating for the low dietary HUFA content. As a result, LA and ALA meet their essential fatty acid (EFA) needs for the ω6 and ω3 families, respectively, although these processes are time-consuming [91,97]. In aquaculture, fish require supplemental PUFA nutrition, particularly C18 and C22 fatty acids, prompting the search for alternative sources of these essential nutrients. PUFAs can be categorized as essential FAs, which animals cannot synthesize de novo and must acquire through diet, or non-essential FAs, which can be synthesized internally [98]. Dietary sources of PUFAs include animals (e.g., fish and seafood), plants, algae, and microorganisms [99]. Beyond their nutritional and health benefits, PUFAs are also utilized in the chemical industry for biofuel production [39].

The synergy between the biological significance of LAs and the versatile capabilities of actinomycetes positions them as a powerful solution in biotechnology. Actinomycetes’ ability to sustainably produce LAs meets the growing demand for essential FAs in aquaculture, human health, and industrial applications. This alignment with environmental and economic sustainability highlights the value of exploring actinomycetes for LA production, driving innovation across diverse sectors.

#### 2.1.4. Omega Families

The FA families ω9, ω7, ω6, and ω5 were identified, with ω9 and ω7 being predominant, while the ω3 family was not detected.

**Figure 4:**
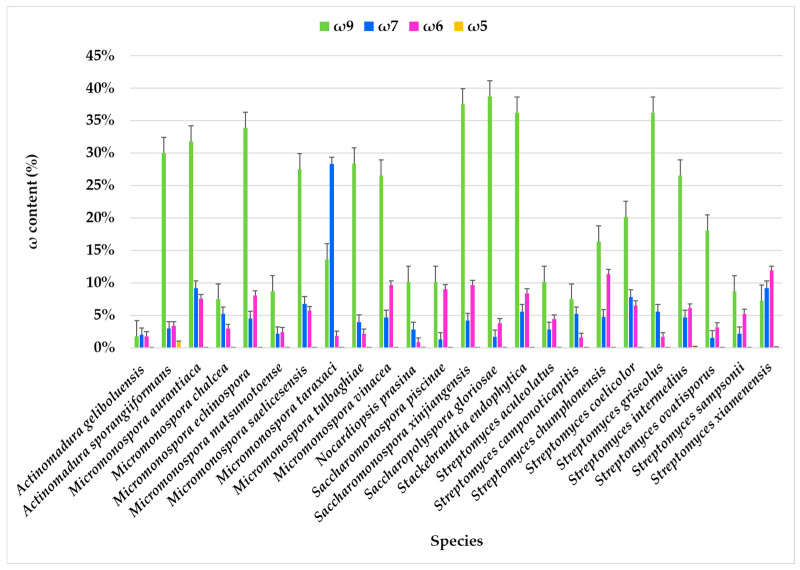
Omega families (ω9, ω7, ω6, and ω5) present in the FAME profile of marine actinobacteria species isolated from marine sediments of the Estremadura Spur.

In most actinomycete species, the ω9 component consistently has the highest content, often reaching or exceeding 25%. The highest expression of ω9 FA was observed in *Nocardiopsis prasina* (38.67%), *Actinomadura sporangiiformans* (37.49%), *Stackebrandtia endophytica* (36.15%), *Micromonospora echinospora* (33.82%), and *Micromonospora aurantiaca* (31.76%). In contrast, the lowest content was found in *Actinomadura geliboluensis*, comprising only 1.73% of the total FAMEs. Comparative analysis of FA profiles across all the species revealed that the ω9 family was present in every species, except for *Streptomyces xiamenensis* and *Micromonospora taraxaci*, where the ω7 family predominated (Figure 4, Table S1). The ω7 content is generally lower than ω9, although it varies significantly. Some species, such as *Micromonospora taraxaci*, *Streptomyces xiamenensis*, *S. chumphonensis*, and *Saccharomonospora xinjiangensis*, showed higher ω7 proportions compared to others. Within these families, C18:1ω9 and C16:1ω7 emerged as the most representative FAs for the ω9 and ω7 families, respectively.

The ω6 family was identified in all species, with the largest abundance in *Streptomyces xiamenensis* (11.90%) and *Streptomyces chumphonensis* (11.34%). In contrast, the lowest expression was observed in *Nocardiopsis prasina*, representing only 0.82% of the total FAMEs. Two ω6 FAs were identified: i-C15:1ω6 and C18:2ω6, with C18:2ω6 being the predominant one. This FA serves as a precursor for other long-chain ω6 FA [10].

The ω5 family was detected exclusively in *Streptomyces xiamenensis* (0.12%), *Streptomyces intermedius* (0.14%), and *Actinomadura sporangiiformans* (0.95%), where C14:1ω5 was the only identified ω5 FA. While rare in actinomycetes, the ω5 family is common in seed oils, particularly pomegranate seeds. The ω5 family is recognized for its potential antioxidant properties [100] and as an anti-proliferative agent, with promising biotechnological applications [101].

The diverse ω9, ω7, ω6, and ω5 FAs found offer significant industrial applications. ω9 FAs, particularly oleic acid, are ideal for cosmetics, biofuels, and food due to their moisturizing, stable, and nutritional properties. ω7 FAs show potential in nutraceuticals, skin health, and biolubricants, while ω6 FAs are valuable for health supplements, anti-inflammatory therapies, and cosmetics. Rare ω5 FAs hold promise for antioxidant products, cancer therapies, and food preservation. Species-specific production and advancements in metabolic engineering and fermentation optimization can enhance their industrial viability, making these actinomycetes a sustainable resource for biotechnology.

#### 2.1.5. Branched-Chain Fatty Acids (BCFAs)

The results revealed that BCFAs were the predominant FAs in the lipid extracts of the actinomycetes strains, accounting for an average of 47.54% of the total identified FAMEs. This outcome was expected, as BCFAs are characteristic of bacteria [41], particularly actinomycetes. Actinomycetes are among the 10% of Gram-positive bacteria known to produce BCFAs [102].

**Figure 5:**
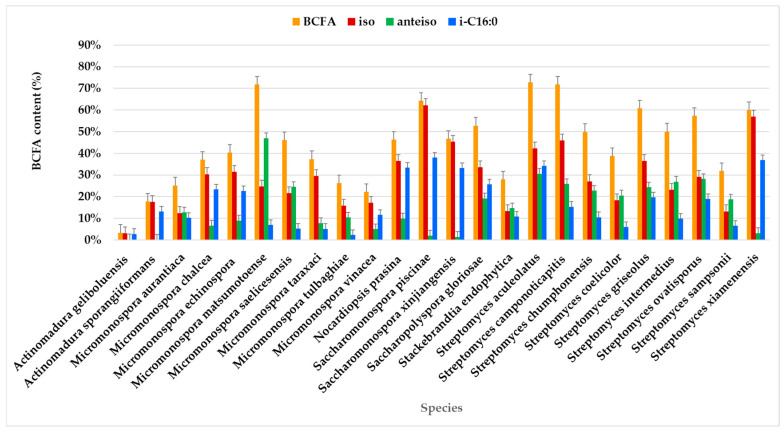
BCFAs comprising the FAME profile identified in the actinomycetes species.

The highest BCFA expression, exceeding 70% of the total FAMEs, was observed in seven strains: *Micromonospora saelicesensis* PTE-083 (83.10%), two *Streptomyces xiamenensis* strains PTE-063 (75.91%) and PTE-065 (72.99%), *Streptomyces aculeolatus* PTE-042 (72.69%), *Micromonospora calchea* PTE-076 (72.60%), *Saccharomonospora gloriosae* PTE-075 (72.40%), and *Micromonospora matsumotoense* PTE-082 (71.71%) (Figure 5, Table S1). In contrast, BCFA content was less abundant in *Actinomadura geliboluensis* (3.34%), representing an unusually low percentage for this species compared to previously reported contents [49].

BCFAs were predominant in most species, with a prevalence of iso-branching (Figure 5). Iso-branching with an even carbon number was particularly predominant. Less common BCFAs, such as i-C15:0 and i-C15:1ω6, were identified in relatively small percentages. Particularly, i-C15:0 is a marker for Gram-positive bacteria [69].

Anteiso branching was exclusively identified in FAs with an odd number of carbon atoms, which aligns with previously reported findings [41]. For example, a-C16:0 has been identified in actinomycetes, including *Aquipuribacter* [103], *Streptomyces* [104,105,106,107], and *Marisediminicola* [108]. Additionally, a-C14:0 has been reported in *Streptomyces* [106,109] and *Nocardioides* [110,111].

Among the identified BCFAs, i-C16:0 predominated in most species, followed by a-C15:0. i-C16:0 was present in all species, though its percentage varied. The highest content of i-C16:0 (≥30%) was observed in *Sacchromonospora piscinae* PTE-081 (38.01%), *Streptomyces xiamenensis* PTE-018 (36.90%), *Streptomyces aculeolatus* PTE-042 (34.17%), *Nocardiopsis prasina* PTE-048 (33.31%), and *Saccharomonospora xinjiangensis* PTE-085 (33.10%) of the total BCFAs. In contrast, the lowest contents of i-C16:0 were found in *Micromonospora tulbaghiae* (2.37%) and *Actinomadura geliboluensis* (2.79%) of the total BCFAs. Interestingly, this contrasts with a previous study where i-C16:0 was reported as the main fatty acid in *Actinomadura geliboluensis*, comprising 19.93% of the total FAMEs [49]. Consistent with the current study, i-C16:0 has been identified as the primary component of *Saccharomonospora piscinae* [112] and *Streptomyces xiamenensis* [113], isolated from fishpond sediments and mangrove environments, respectively.

Regarding a-C15:0, this FA was present in all species, although in varying amounts, with the highest percentage in *Micromonospora matsumotoense* (42.70%) and the lowest in *Saccharomonospora piscinae* (0.61%). The BCFAs with the lowest representation in the studied actinomycetes profiles were i-C17:0 (1.42%), i-C15:1ω6 (2.19%), and i-C14:0 (4.06%). In particular, i-C15:1ω6 was the only unsaturated BCFA identified, with its highest expression in *Streptomyces xiamenensis*. This FA was found in all *Streptomyces* species, except for *Streptomyces camponoticapitis* and *Streptomyces griseolus*. Moreover, the thermophilic actinomycete *Thermobifida fusca* is known to produce a mixture of linear and BCFAs, including iso-, anteiso-, and 10-methyl BCFAs, with chain lengths ranging from 14 to 18 carbons [114].

BCFAs are synthesized from branched-chain amino acids, specifically leucine, isoleucine, and valine, through a decarboxylation and elongation mechanism. This process produces iso-BCFAs with odd and even carbon chains and anteiso-BCFAs with odd carbon chains [42]. The high level of BCFAs produced by the actinomycete strains in this study suggest the potential for pharmaceutical applications, biofuel production, lubricants, and other industrial applications [115].

BCFAs exhibit properties similar to ω3 PUFAs [116]. They have been linked to reductions in type 2 diabetes and obesity [117]. Mika et al. (2016) analyzed serum FA profiles of obese individuals and normal-weight individuals, observing significantly lower BCFA concentrations in obese individuals [116]. Similarly, recent studies confirmed a negative correlation between BCFA levels and obesity [118]. Furthermore, BCFAs may play a role in reducing inflammation. For example, i-C16:0 and i-C18:0 have been shown to inhibit the expression of interleukin-8 (IL-8), a key mediator in inflammation that facilitates neutrophil recruitment and degranulation [119].

Given their beneficial properties, BCFAs have potential applications in pharmaceuticals and cosmetics, particularly in the production of hair and skincare products [42]. Their low melting point [41], excellent oxidative stability, and lubricity make them ideal for producing high-quality biofuels. FAs with chain lengths between C12 and C18 are especially advantageous for biofuel applications due to their lower melting points [120] and enhanced utility in biofuel production [121]. Additionally, BCFAs have uses in industrial applications, such as the production of lubricants, plasticizers, printing inks, and detergents [115].

The FA content in various actinomycete species identified in the present study compared with those reported in the literature is presented in Table 2, highlighting the FAs i-C16:0, C18:1ω9, C16:0, and a-C15:0.

For the FA i-C16:0 (iso-hexadecanoic acid), *Streptomyces xiamenensis* displayed variability, with content measured at 43.82% in the present study compared to 28.40% in a previous report by Takahashi et al. [122]. The highest recorded level of i-C16:0 was found in *Sciscionella sediminilitoris* at 59.50% [123], followed by *Nonomuraea corallina* at 40.40% [124]. Lower levels were noted in *Streptomyces spiramenti* and *Actinoplanes maris*, where i-C16:0 content exceeded 10.00% and 5.00%, respectively. The present study also recorded significant i-C16:0 levels in *Saccharomonospora piscinae* (38.01%) and *Streptomyces xiamenensis* (36.90%).

For C18:1ω9 (oleic acid), differences were observed in *Stackebrandtia endophytica*, which had 36.15% in the present study compared to 2.40% reported by Liu et al. [60]. Similarly, *Nocardiopsis prasina* showed an increase in C18:1ω9 content in the present study (34.83%) compared to the 4.40% recorded by Chen et al. [131].

Regarding C16:0 (palmitic acid), *Actinomadura geliboluensis* showed consistent values, with 24.64% and 22.03% recorded in the present study. Earlier studies, including Sazak et al. (2012) [49] and Zhao et al. (2015) [51], reported lower levels of 13.28% and 21.20%, respectively. These variations suggest species-specific differences in fatty acid biosynthesis under different environmental or experimental conditions.

For a-C15:0 (anteiso-pentadecanoic acid), the present study recorded the highest content in *Saccharomonospora xinjiangensis* at 75.50%, significantly higher than the 5.91% reported by Liu et al. [133]. Other notable levels include 44.80% in *Nesterenkonia marinintestina* [132] and 46.04% in *Brachybacterium atlanticum* [127]. Moderate levels were observed in *Streptomyces pacificus* (15.00%) and *Streptomyces marispadix* (17.70%).

The data revealed substantial interspecies and intra-study variability in FA profiles, reflecting differences in metabolic pathways, environmental influences, and experimental methodologies. The present study adds significant insights, particularly for species such as *Saccharomonospora xinjiangensis*, *Saccharomonospora piscinae*, and *Stackebrandtia endophytica*.

The actinomycete strains in this study produced significant levels of branched-chain fatty acids (BCFAs), with high BCFA content observed in strains like *Micromonospora saelicesensis* and *Streptomyces xiamenensis*. These BCFAs, particularly iso- and anteiso-forms such as i-C16:0 and a-C15:0, offer industrial potential due to their low melting points, excellent oxidative stability, and lubricating properties. This makes them suitable for biofuel production, pharmaceuticals, cosmetics, and industrial products like lubricants and detergents. Their anti-inflammatory and health benefits further enhance their appeal, and the variation in BCFA profiles across strains suggests diverse applications in various industries.

#### 2.1.6. Cyclic Chain Fatty Acids (CCFAs)

Benzene-butanoic acid (benzene-C4:0) was the only FA with a cyclic chain tentatively identified in the present study. It was detected in *Micromonospora tulbaghiae* (9.40%), *Micromonospora vinacea* (5.56%), *Micromonospora saelicesensis* (2.03%), *Micromonospora chalcea* (1.09%), and *Streptomyces griseolus* (0.04%). According to the literature, this FA is uncommon in actinomycetes. However, its identification probability in the NIST library was very high. It has been previously reported in only one study, where it was identified in *Streptomyces* isolated from a marine region of Saudi Arabia, although in a low proportion (3.37%) [134].

Benzene-butanoic acid, also known as 4-phenylbutyric acid or benzene butyric acid, is a phenolic compound comprising an aromatic ring and a carboxylic group. Phenolic compounds are well-known as antioxidant agents [135]. Several studies demonstrated the therapeutic potential of benzene-butanoic acid in supporting the function of vital organs. It is a potent agent in regulating and inhibiting endoplasmic reticulum (ER) stress, a critical process in the management of various diseases, including neurodegenerative conditions [136,137,138,139,140]. Additionally, research has shown that benzene-butanoic acid plays a role in regulating obesity [141], and type 2 diabetes mellitus [136,142].

The detection of benzene-C4:0 in several actinomycete strains, particularly *Micromonospora* species and *Streptomyces griseolus*, highlights its potential industrial applications. This compound, known for its antioxidant properties, has therapeutic applications. These findings suggest that benzene-C4:0 could have valuable applications in pharmaceuticals, nutraceuticals, cosmetics, and food preservation. The discovery of this compound in actinomycetes opens new possibilities for biotechnological use in various industries.

#### 2.1.7. Odd Chain Fatty Acids (OCFAs)

OCFAs are rare in nature but have been identified in plants, animals, fungi, bacteria, and other organisms. They are synthesized through the propionyl-CoA pathway or via the α-oxidation of even-chain FAs [143]. On average, the OCFA content in actinomycete lipid extracts was notably high, comprising 26.93% of the total FA profile, with BCFAs being the majority. However, it was less abundant (<10%) in the strains PTE-033 (0.57%), PTE-046 (2.34%), PTE-051 (2.50%), PTE-078 (2.50%), and PTE-038 (4.59%). The highest OCFA content was observed in the strain PTE-083 belonging to *Micromonospora saelicesensis* and *Micromonospora matsumotoense*, where OCFAs constituted 76.58% and 63.43% of the total FA profile, respectively. Differences in OCFA levels were also noted between the PTE-038 and PTE-083 samples, despite both belonging to the same species. OCFAs were present in all analyzed species, although in a low percentage in *Actinomadura geliboluensis* (0.57%) and *Actinomadura sporangiiformans* (2.34%). Among the identified OCFAs, a-C15:0 was the most prevalent, while C19:1ω9, C17:1ω7, and C15:0 were less abundant (<1%). C15:0 (0.27%) was the least abundant OCFA, detected in all species except *Micromonospora aurantiaca*. In a previous study, C17:1ω7 and C15:0 were identified as major FA components in *Rhodococcus* [143]. *Actinomadura geliboluensis*, isolated from a soil sample in Çanakkale, Turkey, was reported to produce C15:0 (11.60%) [49], which is higher than the content observed in the current study. C15:0 has demonstrated antifungal activity with low minimum inhibitory concentration (MIC). Its mechanism of action involves penetrating the fungal cell membrane, leading to the release of intracellular components, disruption of cellular functions, and ultimately cell death [14]. Besides antifungal properties, OCFAs have garnered industrial interest due to their anti-inflammatory and antiallergic activities [144].

Research suggests that OCFAs, particularly C15:0 and C17:0, are associated with a reduced risk of incidence of type 2 diabetes and cardiovascular diseases [145,146], making them valuable biomarkers for these conditions [11]. Additionally, OCFAs may reduce risk of other chronic diseases by interacting with metabolic regulators, restoring mitochondrial function, and reducing the production of reactive oxygen species (ROS) [146,147]. A recent study by Venn-Watson et al., highlighted the anti-inflammatory and anti-fibrotic activities of C15:0 and C17:0, showing that C15:0 not only mitigates inflammation but also alleviates hemolytic anemia and liver diseases, including severe liver fibrosis and non-alcoholic steatohepatitis (NASH) [146].

OCFAs have broad industrial applications, including use in the pharmaceutical, food, and cosmetic industries for the treatment of psoriasis, allergies, and autoimmune diseases [11]. They are also employed in the chemical industry for the production of pesticides, fragrances, plasticizers, and hydraulic fluids [43].

The actinomycete strains in this study showed high levels of OCFAs, particularly in *Micromonospora saelicesensis* PTE-083 and *Micromonospora matsumotoense*. OCFAs, especially C15:0 and C17:0, offer significant prospects for health applications. Due to these properties, OCFAs have strong potential for use in the pharmaceuticals, cosmetics, food, and chemical industries, making them valuable for therapeutic, skincare, and industrial applications.

### 2.2. Analysis of FAME Profiles of Actinomycete Genera Using GC/MS

**Figure 6:**
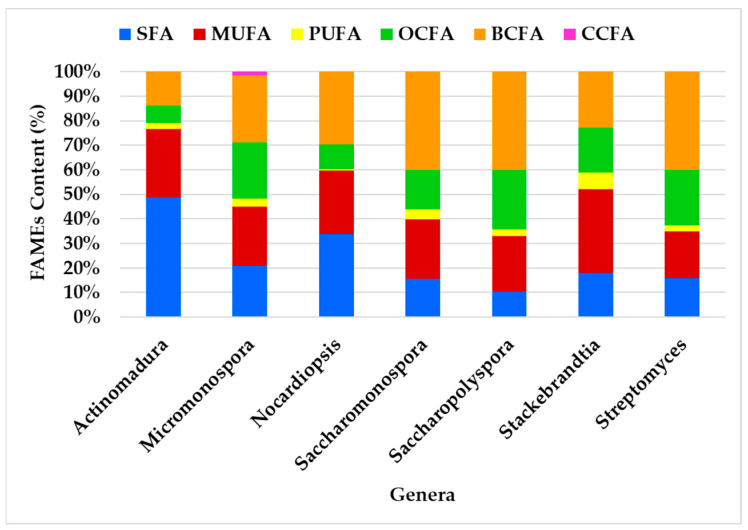
Profiling of the FA classes produced by the actinomycetes genera.

The Estremadura Spur actinomycetes analyzed in this study belong to seven genera: *Actinomadura*, *Micromonospora*, *Nocardiopsis*, *Saccharopolyspora*, *Saccharomonospora*, *Stackebrandtia*, and *Streptomyces*. GC-MS analysis enabled the characterization of the FA class profiles in the ethyl acetate lipid extracts of these actinomycete genera (Figure 6).

The FA class profiles vary among genera, with the most abundant FA being BCFA (41%), followed by MUFA (33%) and SFA (30%). BCFA was the most abundant FA in all genera except *Actinomadura*, where SFA predominates, and *Stackebrandtia*, where MUFA is most prevalent. The genera *Streptomyces*, *Saccharopolyspora*, and *Saccharomonospora* exhibited the most similar profiles (Figure 6). Despite some variability in FAME content among the genera, the overall distribution remains consistent. For instance, BCFAs and SFAs were identified as having the largest content, while OCFAs and MUFAs contribute significantly but to a lesser extent. OCFAs were identified across all genera, but they do not dominate in any of them. PUFAs are the minor fatty acids in all genera. CCFAs were exclusively observed in the *Micromonospora* genus.

**Figure 7:**
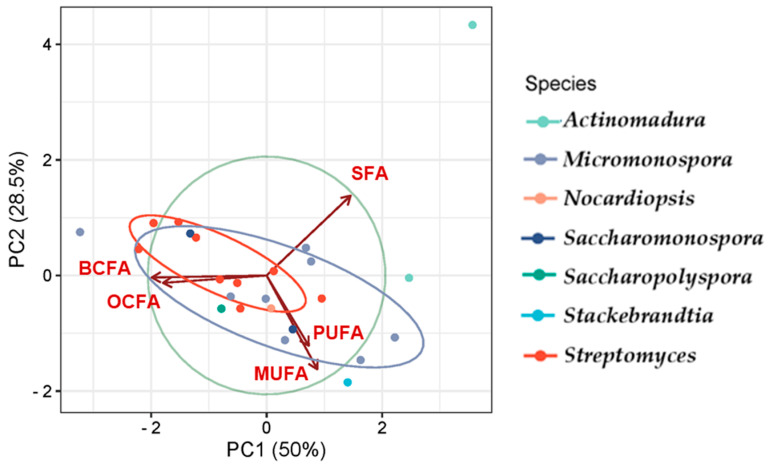
PCA illustrating the FA classes profile according to the actinomycetes genera. The colors correspond to the genera of the actinomycetes.

Principal Component Analysis (PCA) [148] was used to analyze the FA class profiles produced by the actinomycetes and to visualize the variation in FA production across genera (Figure 7). The first two components, PC1 and PC2, explain 78.5% of the variability in the dataset. The PCA results effectively highlight the relationships among the studied genera, revealing distinct clustering patterns that reflect differences in their metabolic, compositional, or genetic characteristics, with overlaps pointing to shared traits or evolutionary relationships.

Genera such as *Streptomyces*, *Saccharopolyspora*, and *Saccharomonospora* presented the greatest similarity in FA production, showing overlap to some extent, which corroborates the observations in Figure 6, and demonstrates the prevalence of BCFAs, MUFAs, and SFAs. *Actinomadura* is presented as an outliner, likely exhibiting unique features, suggesting it is more distinct compared to the others. Variables such as SFA show a strong influence along PC1, while BCFA and MUFA influence both PC1 and PC2. SFAs prevail in the genus *Actinomadura*, while the genus *Stackebrandtia* was the highest MUFA producer.

Actinomycetes, particularly species from the *Streptomyces* genus, are highly valued for their adaptability and efficiency in fermentation processes [149,150], making them ideal candidates for large-scale biotechnological applications. Their well-established use in fermenters can be seamlessly extended to marine-derived strains with minimal adjustments, exploiting their natural resilience to extreme environmental conditions. This resilience not only enhances their tolerance to industrial fermentation processes but also contributes to their ability to produce bioactive compounds, such as FAs. Additionally, actinomycetes are a renewable microbial resource, offering sustainable production of valuable bioactive compounds, which aligns with the principles of the circular bioeconomy. The feasibility of using industrial wastes and seawater as a culture medium further highlights their environmental and economic advantages, reducing the reliance on freshwater resources and contributing to sustainable practices. Furthermore, the genetic tractability of actinomycetes enables precise manipulation to optimize the production of target fatty acids or generate novel derivatives, further enhancing their potential for diverse biotechnological applications.

## 3. Materials and Methods

### 3.1. Ocean Sediments Collection, Strain Taxonomic Characterization and Crude Extract Preparation

The actinomycete strains analyzed in this study were isolated from marine sediments collected off the Portuguese continental coast, specifically from the Estremadura Spur pockmarks field at depths from 200 to 350 m. The samples were obtained using a Smith McIntyre Grab between 31 May and 5 June 2017, as detailed in Pinto-Almeida et al. [21]. The isolation and taxonomic characterization of these strains are also documented in the same study [21], with corresponding GenBank Access IDs: MT830756, MT830767, MT830768, MT830771, MT830775, MT830781, MT830782, MT830784, MT830786, MT830787, MT830790, MT830791, MT830792, MT830793, MT830794, MT830795, MT830796, MT830797, MT830799, MT830800, MT830801, MT830802, MT830803, MT830804, MT830805, MT830806, MT830807, MT830808, MT830809, MT830810, MT830811, MT830812, MT830813, MT830814, MT830815, MT830816, MT830817, MT830818, MT830819, MT830820, MT830821, MT830822, MT830823, MT830824, MT830825, MT830826, MT830827, MT830828, MT830829, MT830830, MT830831, MT830832, MT830833, MT830834.

All isolated actinomycete strains were cultivated under consistent growth conditions as described in reference [21]. Seed cultures of 20 mL were prepared in 100 mL flasks containing medium A1, which consisted of 18 g of agar, 10 g of starch, 4 g of yeast extract, and 2 g of peptone, prepared with filtered seawater and deionized water in a 75:25 (*v*/*v*) ratio. These cultures were incubated for 7 days. Following this, each strain was inoculated into 1 L of medium A1 and cultured with shaking at 220 rpm at 30 °C for 7 days. The resulting culture broth was extracted with EtOAc, and the organic phase was concentrated to dryness under vacuum. Of the strains tested, 54 produced lipidic (oil) crude extracts, which were used for FAME profiling analysis in this study.

### 3.2. Saponification and Derivatization of Crude Actinomycetes Extracts’ Fatty Acids in FAMEs for GC/MS Analysis

FAs present in the oily crude extracts of 54 actinomycetes strains were converted into FAMEs according to the Morrison and Smith protocol with minor modifications [151]; 50 mg of oil was placed in glass tubes, followed by 1 mL of methanolic NaOH solution (0.5 N) (2 g of NaOH/100mL of methanol). Saponification took place during 15 min at 70 °C. After cooling at room temperature, 1 mL of derivatizing agent (BF_3_ in 14% methanol) (14 g of BF_3_/L of methanol, Merk-Schuchardt, Germany) was added and methylation occurred for 10 min at 70 °C. Subsequently, 3 mL of deionized water was added (removing excess BF_3_ and forming an aqueous solution). The next step was the extraction of FAMEs by adding 2 mL of petroleum ether. Then, the samples were vigorously mixed on a vortex for approximately 5 s and the organic phase (supernatant) was transferred to duly identified vials, for analysis by GC/MS.

### 3.3. Analysis of FAMEs by GC/MS

The analysis of the extracted FAMEs was performed using a fully equipped and programmed GC/MS system. The system consists of a GC (Bruker, 456-GC) coupled with mass spectrometer Bruker Scion TQ (Triple Quadrupole, Bremen, Germany), a separation column (DB-WAX Plus, 60 m × 0.32 mm i.d., film thickness 1.0 µm, supplied by Phenomenex, Torrance, CA, USA) and operating with a CTC CombiPal autosampler (Zwingen, Switzerland). The injection was 1 µL of the organic phase using the splitless injection mode. The chromatographic conditions were as follows: temperature 270 °C for the injector, 240 °C for the MS interface, 220 °C for the MS source. Initially, the oven temperature was maintained at 120 °C for 5 min, and subsequently increased from 120 to 250 °C at a rate of 5 °C/min, and finally, 250 °C was maintained for 59 min. He was used as a running gas with a constant pressure of 33 psi (front injector) and 23 psi (central injector), which transports the samples in the gaseous phase with a constant flow of 2.0 mL/min. Ion fragments were obtained by 70 eV electron impact and detected in a *m*/*z* scan = 40 to 450 Da. The standards used were 37-component FAME MIX and 26-component BAME (Supelco). Data were acquired with the Bruker MSWS 8.2 program and analyzed with the Bruker MS Data Review 8.0 software, while the *NIST* library was used for the identification of FAMEs by comparing mass spectra.

### 3.4. Statistical Analysis

Statistical analysis of the results was used to study the correlation among the fatty acid profile and the species of actinomycetes by applying Principal Component Analysis (PCA) using PAST version 4.03 software. The correlation coefficient among FA profile and the different actinomycete species was also determined through Pearson’s correlation using the same software. Statistical significance was given assuming *p* value < 0.05.

## 4. Conclusions

This study presents a comprehensive profiling of FAMEs derived from lipid extracts of actinomycetes isolated from marine sediments from cold seep extreme marine environments, using GC-MS analysis. By investigating 54 strains isolated from Estremadura Spur sediments, focusing on pockmarks’ unique and extreme ecosystems, this work highlights the potential of underexplored marine biodiversity to yield novel biochemical insights. The findings underline the metabolic versatility of actinomycetes, with species-specific dominance of FA types, such as BCFAs, MUFAs, and SFAs, with chain lengths between C12:0 and C20:0. Genera such as *Actinomadura* exhibited SFA dominance, while *Stackebrandtia* emerged as the highest MUFA producer, offering avenues for targeted industrial applications. These compounds exhibit broad biotechnological applications, from antifungal agents in food preservation to precursors for pharmaceuticals, cosmetics, and biofuels. The discovery of dominant SFA (e.g., C18:0 in *Actinomadura geliboluensis*) and high MUFA levels (e.g., C18:1ω9 in *Stackebrandtia endophytica*) highlights their industrial potential. Furthermore, the identification of bioactive compounds like oleic acid (C18:1ω9) and palmitoleic acid (C16:1ω7) with health-promoting properties emphasizes the pharmaceutical relevance of these marine-derived lipids. The identification of unique FA classes such as BCFA, OCFA, and CCFA provides a foundational chemotaxonomic and metabolic framework for future research. The identification of iso- and anteiso-branched FAs, alongside rare FAs like benzene-C4:0, showcases the remarkable biochemical diversity of actinomycetes. This acid was identified in considerable quantities (e.g., 9.40% in *Micromonospora tulbaghiae*), marking a significant biochemical discovery. Exceptional OCFA production in *Micromonospora saelicesensis* (76.58%) highlights its industrial and therapeutic potential. Variations in i-C16:0 and a-C15:0 levels across species provide valuable chemotaxonomic markers for actinomycetes, exemplifying their metabolic versatility and potential for species-specific applications. The observed intra-species metabolic variations, even under identical experimental conditions, provide a chemotaxonomic fingerprint and deepen our understanding of the metabolic plasticity in actinomycetes. Altogether, the actinomycete strains used in this study demonstrate significant biotechnological potential due to their ability to produce various FAs with wide industrial applications. SFAs like palmitic and stearic acids are valuable for biofuels, food preservation, and antifungal formulations, while myristic acid shows promise in combating pathogens. The high levels of MUFAs, such as oleic and palmitoleic acids, offer potential in pharmaceuticals, cosmetics, and food. Rare MUFAs have therapeutic uses in autoimmune diseases and skin care. The detection of benzene-C4:0 and OCFA expands their potential in pharmaceuticals, nutraceuticals, and food preservation. Depending on the biotechnological application goals, the top strains that can be suggested for future FA production scale-up are (1) PTE-057, PTE-082 and PTE-083 with highest BCFA content ≥ 70%; (2) PTE-038, PTE-051, PTE-078, PTE-079, and PTE-086 with highest MUFA content ≥ 40%; (3) PTE-057, PTE-082, and PTE-083 with highest OCFA content ≥ 40%; and (4) PTE-038, PTE-051, PTE-078, PTE-079, and PTE-086 with highest PUFA content ≥ 6%.

This study significantly advances our understanding of FA profiles in marine-derived actinomycetes, bridging the gap between microbial ecology and biotechnology, by linking their metabolic capabilities to applications in health, industry, and environmental sustainability. Our findings not only expand the repository of FAs but also set the stage for the sustainable exploitation of marine microbial resources in industrial applications. Several actinomycetes used in this study can be suggested as cell factories for potential industrial uses in the fields of pharmaceuticals, cosmetics, and other biotechnological applications. Actinomycetes, particularly marine-derived strains, are highly adaptable and efficient for large-scale biotechnological applications. Their resilience to extreme conditions supports industrial fermentation and high-yield production of bioactive compounds like FA. As a renewable resource, actinomycetes enable sustainable production of bioactive compounds, aligning with circular bioeconomy principles and eco-friendly practices. Their ability to utilize seawater and industrial waste as culture media enhances their environmental and economic advantages. Additionally, their genetic flexibility allows for the optimization of FA production and the creation of novel derivatives. Actinomycetes excel in fermentation and are ideal for diverse industrial applications, representing a promising resource for the sustainable production of bioactive FAs, making them valuable for a wide range of biotechnological uses.

## Figures and Tables

**Table 1:** Biotechnological applications of fatty acids.

Fatty Acids	Applications	References
SFA	Biofuel, food industry	[34,35]
MUFA	Cosmetics, biofuel, pharmaceutical, biomedicine	[35,36,37]
PUFA	Aquaculture, pharmaceutical, nutraceutical, cosmetics, biofuel	[33,38,39,40]
BCFA	Biofuel, cosmetics	[41,42]
OCFA	Cosmetics, pharmaceutical, food, nutraceutical, chemistry	[11,43]

**Table 2:** Comparison of the profiles of the most abundant fatty acids identified in the present study and in reported studies.

Fatty Acid	Content (%)	Species	References
i-C16:0	43.82	*Streptomyces xiamenensis*	[113]
	28.40	*Streptomyces pacificus*	[122]
	59.50	*Sciscionella sediminilitoris*	[123]
	40.40	*Nonomuraea corallina*	[124]
	16.00	*Streptomyces yaizuensis*	[125]
	>10.00	*Streptomyces spiramenti*	[126]
	13.70	*Brachybacterium atlanticum*	[127]
	>5.00	*Actinoplanes maris*	[128]
	26.90	*Streptomyces marispadix*	[129]
	>5.00	*Phytohabitans maris*	[130]
	38.01	*Saccharomonospora piscinae*	Present study
	36.90	*Streptomyces xiamenensis*	Present study
C18:1ω9	36.15	*Stackebrandtia endophytica*	Present study
	2.40	*Stackebrandtia endophytica*	[60]
	34.83	*Nocardiopsis prasina*	Present study
	4.40	*Nocardiopsis prasina*	[131]
C16:0	24.64	*Actinomadura geliboluensis*	Present study
	22.03	*Actinomadura geliboluensis*	Present study
	21.20	*Actinomadura sporangiiformans*	[51]
	13.28	*Actinomadura geliboluensis*	[49]
a-C15:0	15.00	*Streptomyces pacificus*	[122]
	75.50	*Saccharomonospora xinjiangensis*	Present study
	44.80	*Nesterenkonia marinintestina*	[132]
	46.04	*Brachybacterium atlanticum*	[127]
	17.70	*Streptomyces marispadix*	[129]
	5.91	*Saccharomonospora xinjiangensis*	[133]

## Data Availability

The data presented in this study are available on request from the corresponding author.

## Supplementary Materials

The following supporting information can be downloaded at: https://www.mdpi.com/article/10.3390/md23010001/s1, Figure S1: Fatty acid profile of the lipidic extract, obtained using ethyl acetate, of the marine-derived actinomycete strain PTE-072, *Saccharomonospora xinjiangensis*, isolated from the Estremadura Spur pockmarks’ oceanic sediments; Table S1: Composition of fatty acids and omega families (%) identified in the ethyl acetate lipid extracts of each actinomycete strain. Letters: M.—*Micromonospora*, S.—*Streptomyces*, Sy.—*Saccharopolyspora*, A.—*Actinomadura*, N.—*Nocardiopsis*, Sac.—*Saccharomonospora*, St.—*Stackebrandtia*, and n.i.—non identified; Table S2: Calculated LRI in DB-WAX Plus, 60 m × 0.32 mm, i.d., film thickness 1.0 µm, supplied by Phenomenex, Torrance, CA, USA.
