# Supplementary material for: GC/MS Fatty Acid Profile of Marine-Derived Actinomycetes from Extreme Environments: Chemotaxonomic Insights and Biotechnological Potential
Source: Mar Drugs. 2024 Dec 24;23(1):1. doi: 10.3390/md23010001 (PMC11767043; doi:10.3390/md23010001)
Supplement: Supplementary file 1 [file marinedrugs-23-00001-s001.zip › marinedrugs-3376716-supplementary.pdf]

marine drugs

MDPI

Supplementary Materials

GC/MS Fatty Acid Profile of Marine-derived Actinomycetes from Extreme Environments: Chemotaxonomic Insights and Biotechnological Potential

Marlene B. Cunha[1,2,3], André F. Jorge [3,4], Maria João Nunes[3], Joana R. Sousa[1,2], Maria João Lança[4], Marco Gomes da Silva[3], Susana P. Gaudêncio[1,2,*]

[1] Associate Laboratory i4HB – Institute for Health and Bioeconomy, NOVA School of Science and Technology, Universidade NOVA de Lisboa, 2829-516 Caparica, Portugal.
[2] UCIBIO-Applied Molecular Biosciences Unit, Chemistry and Life Science Departments, NOVA School of Science and Technology, Universidade NOVA de Lisboa, 2829-516 Caparica, Portugal.
[3] LAQV-Requimte and Department of Chemistry, NOVA School of Science and Technology, Universidade NOVA de Lisboa, 2829-516 Caparica, Portugal.
[4] MED – Mediterranean Institute for Agriculture, Environment and Development & CHANGE – Global Change and Sustainability Institute, Instituto de Investigação e Formação Avançada, Universidade de Évora, Pólo da Mitra, Ap. 94, 7006-554 Évora, Portugal & Departamento de Zootecnia, Escola de Ciências e Tecnologia, Universidade de Évora, Évora, Portugal
* Correspondence: s.gaudencio@fct.unl.pt: SPG

The distribution of FAMEs was observed in the retention time range between 16-34 min, starting from C12:0 to longer chain fatty acids (C20:0). As an example, Figure S1 illustrates a representative GC/MS chromatogram of the FAMEs profile of the lipid extract of the actinomycete strain PTE-072.

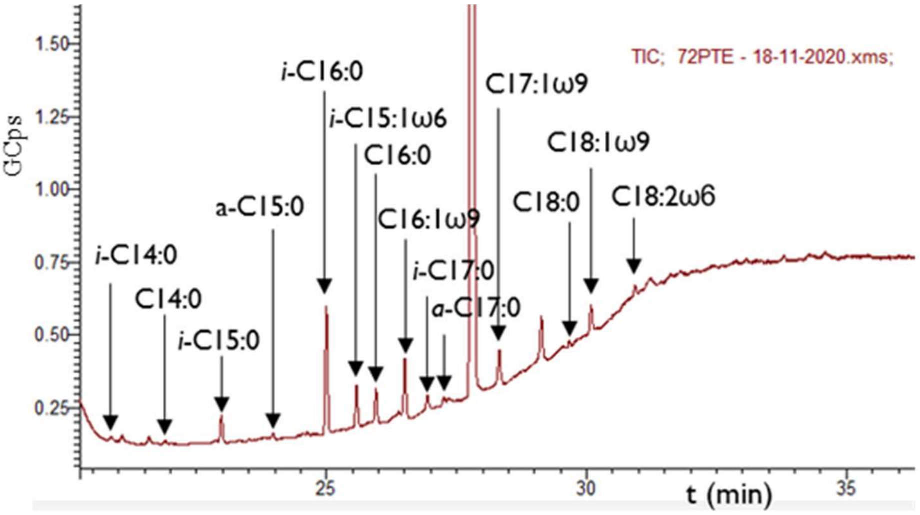

Figure S1. Fatty acids profile of the lipidic extract, obtained using ethyl acetate, of the marine-derived actinomycete strain PTE-072, *Saccharomonospora xinjiangensis*, isolated from the Estremadura Spur pockmarks oceanic sediments.

Table S1. Composition of fatty acids and omega families (%) identified in the ethyl acetate lipid extracts of each actinomycete strain. The letters, *M.- Micromonospora*, *S.- Streptomyces*, *Sy.- Saccharopolyspora*, *A.- Actinomadura*, *N.- Nocardiopsis*, *Sac.- Saccharomonospora*, *St-. Stackebrandtia*, and n.i.- non identified.

Strain	Species	SFA	MUFA	PUFA	BCFA	*iso*	*anteiso*	CCFA	OCFA	ω9	ω7	ω6	ω5
**PTE-007**	*M. vinacea*	31.55	31.15	9.61	22.14	17.15	4.99	5.56	10.16	26.51	4.64	9.61	0.00
**PTE-018**	*S. xiamenensis*	25.89	37.99	5.96	34.10	31.89	2.21	0.00	10.93	21.70	11.66	9.89	0.70
**PTE-019**	*Sy. gloriosae*	15.59	36.62	3.65	44.14	34.11	10.02	0.00	11.28	34.40	2.22	3.65	0.00
**PTE-022**	*M. taraxaci*	22.08	38.76	1.84	37.31	29.49	7.82	0.00	33.06	13.58	25.18	1.84	0.00
**PTE-026**	*S. camponoticapitis*	14.10	12.60	1.55	71.75	45.88	25.86	0.00	30.62	7.43	5.18	1.55	0.00
**PTE-032**	*S. intermedius*	33.01	27.19	6.04	33.75	11.78	21.98	0.00	27.51	18.90	7.74	6.04	0.55
**PTE-033**	*A. geliboluensis*	91.19	3.69	1.78	3.34	3.08	0.26	0.00	0.57	1.73	1.96	1.78	0.00
**PTE-035**	*S. sampsonii*	31.38	32.35	4.73	31.54	8.84	22.70	0.00	28.23	25.55	6.80	4.73	0.00
**PTE-037**	*M. chalcea*	40.56	30.77	2.33	26.34	22.29	4.05	0.00	25.73	14.51	16.27	2.33	0.00
**PTE-038**	*M. saelicesensis*	34.83	42.95	9.10	9.07	7.41	1.66	4.06	4.59	35.10	7.85	9.10	0.00
**PTE-041**	*S. griseolus*	19.93	14.86	1.64	63.57	45.76	17.81	0.00	30.18	9.79	5.07	1.64	0.00
**PTE-042**	*S. aculeolatus*	11.79	14.58	2.65	72.69	42.19	30.51	0.00	38.21	10.16	2.70	4.37	0.00
**PTE-043**	*S. griseolus*	22.62	15.26	1.44	60.68	34.94	25.73	0.00	39.20	7.84	7.42	1.44	0.00
**PTE-044**	*S. intermedius*	27.50	32.40	4.06	39.57	20.50	19.07	0.00	30.97	18.70	10.17	7.60	0.00
**PTE-045**	*S. coelicolor*	30.14	31.38	3.09	38.84	18.29	20.55	0.00	32.45	20.11	7.82	6.53	0.00
**PTE-046**	*A. sporangiiformans*	37.37	43.67	2.17	17.68	17.53	0.16	0.00	2.34	36.93	5.85	3.06	0.00
**PTE-047**	*N. prasina*	12.88	38.05	0.00	49.07	38.69	10.38	0.00	14.55	37.15	0.91	0.00	0.00

**PTE-048**	*N. prasina*	16.84	37.71	0.00	45.45	35.35	10.10	0.00	16.04	35.88	1.83	0.00	0.00
**PTE-050**	*S. sampsonii*	31.28	36.04	4.71	27.97	13.52	14.45	0.00	16.95	29.37	6.67	4.71	0.00
**PTE-051**	*S. sampsonii*	38.36	49.24	7.04	5.37	3.64	1.72	0.00	2.50	41.50	7.73	7.04	0.00
**PTE-052**	*S. griseolus*	17.94	16.97	1.26	63.84	36.58	27.26	0.00	41.58	9.09	7.88	1.26	0.00
**PTE-053**	*S. intermedius*	13.38	21.20	2.43	65.02	30.94	34.08	0.00	54.26	11.10	8.07	4.46	0.00
**PTE-054**	*S. ovatisporus*	21.60	21.09	1.60	57.26	29.14	28.13	0.00	34.90	18.02	1.52	3.15	0.00
**PTE-055**	*M. echinospora*	17.77	42.81	3.56	40.35	31.39	8.96	0.00	25.65	33.82	4.50	8.06	0.00
**PTE-056**	*M. tulbaghiae*	29.98	32.28	2.15	26.20	15.83	10.36	9.40	25.29	28.36	3.93	2.15	0.00
**PTE-057**	*S. chumphonensis*	16.33	17.94	1.43	66.22	38.66	27.57	0.00	49.53	11.76	4.26	3.35	0.00
**PTE-058**	*S. chumphonensis*	30.64	24.40	19.33	25.64	14.11	11.53	0.00	18.93	20.89	3.50	19.33	0.00
**PTE-059**	*S. xiamenensis*	16.50	32.82	0.54	58.49	56.51	1.98	0.00	15.80	4.29	20.19	8.88	0.00
**PTE-060**	*S. griseolus*	25.26	17.49	2.24	54.84	28.50	26.33	0.17	35.26	12.67	4.82	2.24	0.00
**PTE-061**	*S. chumphonensis*	17.64	22.86	1.79	57.71	28.51	29.20	0.00	38.79	16.46	6.40	1.79	0.00
**PTE-062**	*Sy. gloriosae*	17.99	29.20	3.11	49.70	26.03	23.67	0.00	37.95	25.56	3.65	3.11	0.00
**PTE-063**	*S. xiamenensis*	11.80	22.29	0.30	75.91	71.65	4.26	0.00	18.79	2.26	9.73	10.60	0.00
**PTE-064**	*S. sampsonii*	18.21	17.34	3.10	62.61	26.87	35.74	0.00	44.44	10.97	5.11	4.36	0.00
**PTE-065**	*S. xiamenensis*	8.47	36.42	0.25	72.99	72.18	0.81	0.00	22.41	1.98	16.29	18.39	0.00
**PTE-066**	*N. prasina*	8.09	45.13	2.47	44.31	35.23	9.09	0.00	15.67	42.98	2.15	2.47	0.00
**PTE-067**	*Sy. gloriosae*	12.30	32.29	2.68	52.74	28.15	24.59	0.00	45.44	28.02	4.26	2.68	0.00

**PTE-068**	*M. chalcea*	45.76	34.38	4.36	12.23	6.19	6.04	3.28	10.01	29.62	4.76	4.36	0.00
**PTE-069**	*Sac. gloriosae*	11.18	32.90	4.78	51.57	31.17	20.39	0.00	40.10	29.59	2.88	5.21	0.00
**PTE-079**	*S. xiamenensis*	22.92	36.78	1.54	52.41	45.94	6.47	0.00	27.29	7.32	15.82	15.19	0.00
**PTE-071**	*S. intermedius*	15.45	23.75	2.57	61.88	29.53	32.34	0.00	43.72	11.53	8.57	6.22	0.00
**PTE-072**	*Sac. xinjiangensis*	11.62	40.44	2.10	56.21	54.92	1.29	0.00	30.66	15.21	14.87	12.46	0.00
**PTE-073**	*S. xiamenensis*	13.43	26.43	1.24	66.11	63.18	2.94	0.00	14.13	5.93	13.29	8.45	0.00
**PTE-074**	*Sac. xinjiangensis*	16.54	24.31	3.20	59.57	56.59	2.98	0.00	18.30	11.63	9.06	6.83	0.00
**PTE-075**	*Sac. gloriosae*	5.69	20.39	1.52	72.40	53.45	18.95	0.00	34.51	19.18	1.21	1.52	0.00
**PTE-076**	*M. chalcea*	9.26	16.03	2.12	72.60	62.74	9.85	0.00	19.87	14.52	1.51	2.12	0.00
**PTE-077**	*Sac. gloriosae*	18.68	28.74	6.52	46.06	28.68	17.38	0.00	23.98	23.73	5.01	6.52	0.00
**PTE-078**	*Sac. xinjiangensis*	29.19	42.85	13.79	14.16	14.16	0.00	0.00	2.50	29.04	13.81	13.79	0.00
**PTE-079**	*St. endophytica*	21.95	41.70	8.38	27.97	13.29	14.68	0.00	22.21	36.15	5.55	8.38	0.00
**PTE-080**	*A. sporangiiformans*	29.90	42.44	3.64	24.01	14.00	10.01	0.00	19.97	38.04	2.51	3.64	1.89
**PTE-081**	*Sac. piscinae*	22.13	18.15	2.25	64.23	62.20	2.03	0.00	31.69	10.17	1.23	9.00	0.00
**PTE-082**	*M. matsumotoense*	15.09	10.79	2.41	71.71	24.72	46.99	0.00	63.43	8.65	2.13	2.41	0.00
**PTE-083**	*M. saelicesensis*	8.81	5.86	2.23	83.10	35.82	47.28	0.00	76.58	4.87	0.99	2.23	0.00
**PTE-085**	*Sac. xinjiangensis*	17.89	26.20	3.80	56.94	55.47	1.47	0.00	16.81	12.50	8.88	8.61	0.00
**PTE-086**	*M. aurantiaca*	26.37	40.92	7.54	25.16	12.41	12.75	0.00	19.80	31.76	9.16	7.54	0.00
**Average (%)**		22.16	28.49	3.58	47.54	32.42	15.12	0.41	26.93	19.44	6.81	5.77	0.06

Table S2. Calculated LRI in DB-WAX Plus, 60 m x 0.32 mm, i.d., film thickness 1.0 µm, supplied by Phenomenex, Torrance, CA, USA.

FAME's	LRI (Calculated)	Standard (37-component FAMEs MIX and 26-component BAME)
C9:0	1312	No*
C11:0	1519	Yes
C12:0	1622	Yes
C13:0	1727	Yes
C14:0	1830	Yes
C15:0a	1883	Yes
C15:0i	1914	Yes
C15:0	1934	Yes
C16:0i	1987	Yes
C16:0	2038	Yes
C16:1ω9	2064	No*
C16:1ω7	2071	Yes
C16:1ω5	2083	No*
C17:0a	2090	No*
C17:0 i	2108	Yes
C17:0	2139	Yes
C17:1ω7	2170	Yes
C18:0i	2193	No*
C18:0-a	2210	No*
C18:0	2245	Yes
C18:1ω9t	2263	Yes
C18:1ω9	2274	Yes
C18:1ω7	2281	No*
C18:1ω5	2292	No*
C19:0i	2315	No*
C18:2ω6	2323	Yes
C19:0	2345	Yes
C18:3ω6	2357	Yes
C19:1ω9	2377	No*
C18:3ω3	2393	Yes
C18:4ω3	2426	No*
C20:0	2447	Yes
C20:1ω9	2476	Yes
C20:2ω6	2528	Yes
C20:3ω6	2560	Yes
C20:4ω6	2591	Yes
C20:3ω3	2600	Yes

C20:4ω3	2630	No*
C22:0	2652	Yes
C20:5ω3	2657	Yes
C22:1ω9	2680	Yes
C22:2ω6	2736	Yes
C22:4ω6	2795	No*
C22:5ω6	2825	No*
C24:0	2859	Yes
C22:5ω3	2865	No*
C24:1ω9	2891	Yes
C22:6ω3	2900	Yes

** Not co-injected as standard but in the probability region according to (Fish Physiol Biochem, https://doi.org/10.1007/s10695-018-0580-3)*
